# Functional relationship of furfural yields and the hemicellulose-derived sugars in the hydrolysates from corncob by microwave-assisted hydrothermal pretreatment

**DOI:** 10.1186/s13068-015-0314-z

**Published:** 2015-08-27

**Authors:** Huiling Li, Xiaofeng Chen, Junli Ren, Hao Deng, Feng Peng, Runcang Sun

**Affiliations:** State Key Laboratory of Pulp and Paper Engineering, South China University of Technology, Guangzhou, 510640 China; Beijing Key Laboratory of Lignocellulosic Chemistry, Beijing Forestry University, Beijing, 100083 China

**Keywords:** Corncob, Depolymerization of hemicelluloses, Furfural, Two-step process, Microwave-assisted hydrothermal pretreatment, Heterogeneous catalysis

## Abstract

**Background:**

Corncob as one of the most suitable feedstock for the production of a variety of high-value-added chemicals is receiving increasing attention worldwide because of the characteristics of high carbohydrate (cellulose and hemicelluloses) contents and high energy densities. Furfural produced from hemicelluloses is a highly versatile and key feedstock used in the manufacture of a wide range of biofuel and important chemicals in different fields. Achieving high furfural yields from corncob combining green approaches and efficient equipment has the promising potential for biomass-to-biofuel technologies. To understand the dissolving mechanism of corncob sugars and reveal the relationship between the hydrolysate composition and furfural yields, a two-step approach was proposed using microwave-assisted hydrothermal pretreatment and subsequently heterogeneous catalytic process.

**Results:**

Released hemicelluloses in the first stage were mainly in forms of monosaccharide, oligosaccharides, and water-soluble polysaccharide. Hydrolysates with the maximum xylose content (99.94 mg g^−1^, 160 °C, 90 min), the maximum xylobiose content (20.89 mg g^−1^, 180 °C, 15 min), and the maximum total xylose content in monosaccharide and oligosaccharides (DP ≤ 6) (272.06 mg g^−1^, 160 °C, 60 min) were further converted to furfural using tin-loaded montmorillonite as the catalyst in a biphasic system. The highest furfural yield (57.80 %) was obtained at 190 °C for 10 min from hydrolysates with the maximum xylose content. Moreover, controlled experiments showed that furfural yields from corncob hydrolysates were higher than those from the pure xylose solutions, and lower initial xylose concentration may be in favor of the furfural production.

**Conclusions:**

This work provides an efficient approach to produce furfural by a two-step process for the biomass-to-biofuel industry. Results indicated that the production of furfural from biomass raw materials can be controlled by the depolymerization degree of hemicelluloses.

**Electronic supplementary material:**

The online version of this article (doi:10.1186/s13068-015-0314-z) contains supplementary material, which is available to authorized users.

## Background

The depletion of fossil fuel reserves, the increasing environmental and political pressures, and the unstable situation of fuel prices have drawn attention to the lignocellulosic biomass as an alternative resource of petroleum [1]. Agricultural residues, such as corncob, represent a highly sustainable resource of bio-platform molecules and chemicals for significant industrial use [2]. Furthermore, characteristics of high carbohydrate (cellulose and hemicelluloses) contents and high energy densities make corncob as one of the most suitable feedstock for the production of a variety of high-value-added chemicals such as furfural [3, 4].

Furfural is a secondary fuel precursor directly from biomass with an annual production volume of more than 200,000 tons [5]. This versatile intermediate is considered as an important chemical solvent for many organic materials and holds great promise as primary building blocks for the synthesis of a multitude of important non-petroleum-derived chemicals such as furfuryl alcohol, tetrahydrofurfuryl alcohol, methyltetrahydrofuran and polyols through oxidation, hydrogenation, hydrogenolysis and condensation reactions [6, 7]. Furfural is industrially synthesized from pentose-rich lignocellulosic materials using mineral acids as catalysts. However, old and inefficient strategies, equipment corrosion and waste disposal, and low production yields have strongly diminished its competitiveness with petroleum-based alternatives in the global market [8]. Achieving high furfural yields from corncob integrating green approaches and efficient equipment has the promising potential for biomass-to-biofuel technologies.

There is an urgent demand to develop novel effective strategies for overcoming the major drawbacks of industrial furfural production. Solid acid catalysts, which possess properties such as thermal stability and recyclability, are deemed to benefit for the furfural production process [9]. Biphasic systems, which can hinder the second reactions by simultaneously extracting furfural from the aqueous medium to the organic phase, have received much attention [10, 11]. Extensive studies on xylose dehydration have been performed using solid acids as catalysts in biphasic systems [12–14]. However, detailed information on the direct conversion of lignocellulosic biomass to furfural is inadequate. Many natural factors impede the effective conversion of biomass, such as the crystalline domains of cellulose, the structural heterogeneity and the complexity of cell-wall constituents as well as its complex cross-linking [15]. To achieve the good contact between corncob sugars and the solid catalyst, it is necessary to destroy the complicated structure of the solid raw materials first and then obtain as many hydrophilic products, such as soluble polysaccharides, oligosaccharides and monosaccharide, as possible [16]. Further converting these sugars by solid catalysts in biphasic systems would then be desirable. This approach can effectively enhance the carbohydrate conversion rate and overcome the regeneration problem of the solid catalyst arising from the one-step conversion method.

Although the hydrothermal pretreatment (HTP) of the whole biomass has been investigated by various studies previously in the aspect of the composition of different sugars in hydrolysates or residues individually [17–19], only few investigations took a whole view of both hydrolysates and pretreated materials to understand the dissolution and degradation trends of sugars and even put a perspective on the functional relationship between the hydrolysate composition and the high-value-added chemicals production. Unveiling the dissolving trends of hemicellulose-derived sugars from the cell wall of lignocellulose under HTP process will facilitate building on the relationship between operated conditions and the furfural yields. Our previous studies showed that tin-loaded montmorillonite (Sn-MMT) had a great catalytic ability and stable recyclability for the furfural production from xylose, water-insoluble hemicelluloses and water-soluble fraction of corncob in a biphasic system with 2-sec-butylphenol (SBP) as the organic extracting layer and dimethyl sulfoxide (DMSO) as the co-solvent in contact with an aqueous phase saturated with NaCl (SBP/NaCl-DMSO) [20]. In this paper, we demonstrate a novel approach: microwave-assisted HTP of corncob, followed by the heterogeneously catalyzed conversion of the hemicellulose-derived sugars in hydrolysates over Sn-MMT in the SBP/NaCl-DMSO system for the furfural production. This approach has several advantages: uniform and selective heating of microwave, efficient utilization of corncob sugars, the avoidance of corrosion and pollution of mineral acids, as well as the ability of depressing the side reactions [21]. The dissolution and depolymerization of hemicelluloses from corncob by the microwave-assisted HTP were assessed, the optimum microwave-assisted hydrolysis conditions were identified, and the relationship between the hydrolysate composition and the furfural yield was investigated.

## Results and discussion

### The dissolution and depolymerization of hemicelluloses from corncob by microwave-assisted hydrothermal pretreatment

Recent studies showed that hemicelluloses fraction in lignocellulosic materials (LCMs) can be effectively depolymerized into soluble monomers, oligosaccharides, and lower molecular weight polymers during the HTP process [22, 23]. The proportion of the soluble compounds depends on the operation conditions. In the present study, microwave-assisted hydrothermal pretreatments of corncob were performed as the first stage at given temperatures (120–180 °C) for desired periods, and the main components of hydrolysates are shown in Table 1.

**Table 1:** Composition of hydrolysates (mg per gram sample, mg g^−1^) after the microwave-assisted hydrothermal pretreatment

Temp. (°C)	Time (min)	pH	Glucose	Xylose	Arabinose	FA	AA	FF	HMF	Oligosaccharide (DP ≤ 6)					EPH	Xylose content
										X2	X3	X4	X5	X6		
120	0	5.63	3.14	3.23	0.00	0.00	0.79	0.00	0.00	0.00	0.00	0.00	0.00	0.00	0.00	3.23
120	30	5.29	3.12	3.51	1.07	0.00	1.23	0.00	0.00	0.00	0.00	0.00	0.00	0.00	0.00	3.51
120	60	5.25	3.01	3.52	1.16	0.00	2.03	0.00	0.00	0.00	0.00	0.00	0.00	0.00	0.00	3.52
120	90	5.23	2.96	3.54	2.85	0.30	2.77	0.00	0.00	0.66	0.31	0.14	0.33	0.14	21.60	8.82
120	120	5.23	2.93	3.58	3.64	0.33	3.27	0.00	0.00	3.17	2.20	0.26	0.44	0.34	22.90	21.82
140	0	4.63	2.96	3.29	0.34	0.00	1.28	0.00	0.00	0.48	0.00	0.00	0.00	0.00	0.00	4.25
140	30	4.21	2.79	3.77	5.31	0.28	3.10	0.00	0.00	1.11	0.28	0.52	1.03	0.76	76.90	18.62
140	60	4.04	2.78	6.91	7.24	0.51	6.92	0.73	0.00	3.07	2.79	2.23	3.31	1.06	118.00	53.24
140	90	3.88	2.66	10.30	8.23	0.64	8.54	1.56	0.00	6.07	6.11	4.40	2.30	2.29	90.20	83.62
140	120	3.85	1.46	11.87	8.97	0.67	9.92	1.59	0.00	8.87	9.35	7.02	4.60	3.02	72.90	126.85
160	0	4.38	1.75	3.42	3.75	0.25	2.57	0.22	0.00	0.59	0.00	0.00	0.00	0.69	55.60	8.73
160	30	3.70	1.04	18.45	9.72	0.93	8.02	2.73	0.00	14.30	13.05	13.75	3.53	3.76	39.00	181.38
160	60	3.58	2.04	59.21	6.89	1.53	18.30	11.60	1.14	14.69	22.29	15.56	7.78	3.16	0.00	272.06
160	90	3.44	2.81	99.94	6.45	2.13	24.77	26.43	1.71	5.72	19.72	5.86	3.47	2.87	0.00	228.54
160	120	3.40	7.40	81.00	6.43	4.61	33.20	30.80	1.80	4.95	3.22	2.08	3.83	2.99	0.00	146.00
180	0	3.91	1.79	11.23	6.44	0.55	7.01	1.17	0.00	5.13	5.44	3.95	0.47	1.18	114.00	66.82
180	5	3.82	1.60	19.15	7.28	0.62	9.99	3.18	0.00	13.38	13.40	8.20	5.88	3.69	72.10	170.44
180	10	3.65	2.06	22.51	12.91	1.72	12.89	4.35	0.00	16.00	17.52	16.23	7.18	4.38	28.40	234.19
180	15	3.64	2.19	31.07	13.27	1.95	14.08	7.05	1.80	20.89	19.04	16.78	7.25	3.10	0.00	251.96
180	30	3.62	7.39	65.78	4.51	4.40	29.64	27.28	2.36	5.96	19.21	4.72	8.84	2.27	0.00	211.97

Reaction conditions: 2.0 g of corncob in 20 mL of DI water was subjected to microwave heating (600 W)

*FA* formic acid, *AA* acetic acid, *FF* furfural, *HMF* 5-hydroxymethylfurfural, *EPH* ethanol-precipitated hemicelluloses, *Xylose content* the total xylose in monosugar and oligosaccharide (DP ≤ 6)

The pH of hydrolysates decreased as the temperature rose and time prolonged, which was expected to be the reason that part of the acetyl groups present in the hemicelluloses of biomass was cleaved to generate acetic acid [24]. This was also verified by the steady increase of acetic acid content with increasing the reaction temperature and time in Table 1. In addition, furan compounds, such as furfural and 5-hydroxymethylfurfural (HMF), obtained from the further dehydration of the released monosugars during the HTP process, could be degraded to form organic acids, such as formic acid, which also reduce the pH of hydrolysates. The reaction severity for the furfural formation was much more moderate than that for HMF as observed in Table 1, which was ascribed to the higher xylose amount than glucose in hydrolysates because hemicelluloses are generally easier degraded than cellulose. Due to the higher temperature and longer pretreatment time being in favor of the furfural and HMF formation as well as their degradation, the yield of the formic acid was enhanced with the increase of the process severity. As a result, higher acidity was obtained at harsher treatment conditions, which in turn aids in a series of autohydrolysis process [25]. Although furfural was our target product, its yield was low at these given reaction conditions. The objective of our work at the first stage was to obtain as many hydrophilic products (soluble polysaccharide, oligosaccharides and monosaccharide) as possible, which could give the better opportunity to contact different sugars with solid catalysts for the further furfural production at the second stage. Therefore, the hydrolysate composition should be seriously considered and the operation conditions should be carefully selected in the first stage due to the purpose of the high furfural yield and selectivity in the second stage.

**Fig. 1:**
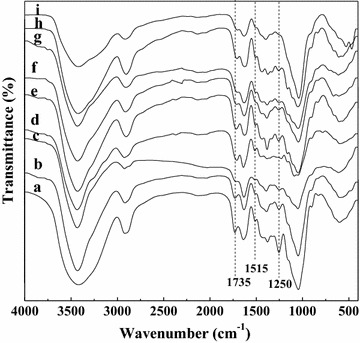
FT-IR spectra of the solid residues obtained after the microwave-assisted hydrothermal pretreatmentHTP: (*a*) raw corncob, (*b*) hydrolysis at 120 °C for 0 min, (*c*) hydrolysis at 140 °C for 0 min, (*d*) hydrolysis at 160 °C for 0 min, (*e*) hydrolysis at 180 °C for 0 min, (*f*) hydrolysis at 160 °C for 30 min, (*g*) hydrolysis at 160 °C for 60 min, (*h*) hydrolysis at 160 °C for 90 min, (*i*) hydrolysis at 160 °C for 120 min

The Fourier-transform infrared (FT-IR) spectra of the raw corncob as well as the hydrothermally treated samples at different temperatures (120, 140, 160 and 180 °C) of constant reaction time (0 min), and different time (0–120 min) at the temperature of 160 °C were subsequently conducted to evaluate the cleavage of acetyl groups in corncob during the HTP process (Fig. 1). Bands around 1735 and 1250 cm^−1^ are mainly ascribed to the stretching vibration of C=O and C–O bonds of the acetyl ester units in hemicelluloses, respectively [26]. The decrease in the intensity of these bands was indicative of the dissolution of hemicelluloses from corncob and the deacetylation of hemicelluloses, which was consistent with the results that the acetic acid content in the hydrolysates enhanced (Table 1) and the weight percentage of xylose in the corncob residues decreased (Table 2) with increasing the pretreated temperature and time. The peak at 1515 cm^−1^ is attributed to the skeletal vibrations of the lignin aromatic rings [27]. Only a slight increase was observed for the intensity of this peak, which was also evident from the increase of the acid-insoluble fraction in the treated corncob analyzed by the NREL method (Table 2).

**Table 2:** Composition of solid residues (weight percentage, wt%) after the microwave-assisted hydrothermal pretreatment

Temp. (^o^C)	Time (min)	Glucose (wt%)	Xylose (wt%)	Arabinose (wt%)	Acid-insoluble fraction (wt%)
Raw material		37.09	31.39	2.28	14.47
120	0	38.07	30.91	2.26	16.47
120	30	38.54	30.77	2.24	16.77
120	60	38.75	30.76	2.22	18.19
120	90	38.87	30.44	1.97	19.38
120	120	39.34	28.79	1.75	20.27
140	0	38.36	29.60	2.22	17.01
140	30	43.91	28.27	1.60	18.34
140	60	51.92	21.91	1.07	19.02
140	90	54.78	18.00	0.75	19.65
140	120	57.25	15.56	0.62	21.44
160	0	41.58	29.07	1.82	17.29
160	30	63.17	13.19	0.57	18.79
160	60	69.82	7.14	0.00	19.72
160	90	70.49	5.35	0.00	20.48
160	120	28.13	1.61	0.00	60.24
180	0	61.57	14.81	0.00	18.60
180	5	62.55	10.76	0.00	19.22
180	10	68.72	6.97	0.00	19.34
180	15	77.40	4.47	0.00	19.51
180	30	50.83	3.14	0.00	39.31

Reaction conditions: 2.0 g of corncob in 20 mL of DI water was subjected to microwave heating (600 W)

The morphological structures of the treated samples are illustrated in Figure S1 (Additional file 1). Substantial changes in the surface morphology and pore structure could be observed after the microwave-assisted HTP. The network structure of the raw material and the regular pore construction were destroyed under harsh conditions. However, there was no obvious change of the pretreated corncob’s crystal structure after the HTP process (Additional file 1: Figure S2).

The process liquids were also analyzed for monosugars, oligosaccharides and water-soluble polysaccharides as described in the “Methods” section, and the results are shown in Table 1. During the HTP under 200 °C, almost all of hemicelluloses and a portion of cellulose can be released from the biomass to form water-soluble polysaccharides and oligosaccharides. These polysaccharides and oligosaccharides can further hydrolyze to produce monosaccharides in particular conditions [28]. Xylose, as the predominant neutral monosaccharide, was present at low productivity (<12 mg g^−1^) under mild conditions (≤140 °C and ≤120 min). Increased xylose monomer yield was achieved through prolonging reaction time or improving reaction temperature, indicating that the degree of the hydrolysis of hemicelluloses was enhanced by the increase of the treatment intensity. However, the yield of xylose decreased under intense conditions (160 °C for 120 min), which was ascribed to the reason that xylose can be further dehydrated to form furan compounds [29]. Note that the xylose quantities reported here are more than sufficient to explain the apparent reduction in the xylose content reported for the analysis of structural carbohydrates in solid residues (Table 2). A maximum yield of xylose was detected at 160 °C for 90 min (99.94 mg g^−1^).

Arabinose was another monosugar in the filtrate obtained from the HTP of corncob. It presents in a form of arabinoxylan in corncob hemicelluloses [30]. The linear *β*-(1,4)-d-xylopyranose backbone is substituted by *α*-l-arabinofuranosyl units in the positions 2-O and/or 3-O [31]. The yield of arabinose increased with the increase of pretreatment time at relative low temperatures (120 and 140 °C). Additionally, it is noteworthy that there was no big gap between the yields of xylose and arabinose in mild conditions, suggesting that the degradation of hemicelluloses side chains and the backbone undergone at the same time. About 39.34 % of arabinose could be obtained from the hydrolysis of hemicelluloses at 140 °C for 120 min, while only 3.78 % of xylose was achieved. This indicates that the hydrolysis of side chains was much easier than that of the backbone, which was resulted from the higher structural angle strains in the furanoside sugar units (arabinose), whereas pyranose rings (xylose) are strain-free [32]. Nearly all the arabinose component in corncob was dissolving out at high treatment severity (after 60 min at 160 °C or the temperature higher than 160 °C, Table 2). However, when the temperature was up to 160 °C, the yield of arabinose in hydrolysates increased first and then decreased with prolonging the pretreatment time, which was ascribed to the side reactions in intense conditions [33]. Moreover, the yield of arabinose was much lower than that of xylose at high temperatures (160 and 180 °C). This observation offered the hypotheses that the cleavage of *β*-(1,4)-d-xylopyranose backbone played the most important role at high temperatures.

Small amounts of glucose were also recovered during the HTP process. According to the literature, it is believed that the glucose was from glucuronoarabinoxylans and xyloglucans in corncob [30]. Low yields of glucose (≤7.40 mg g^−1^) were detected in all process liquids (Table 1), implying that cellulose was more difficult to hydrolyse than hemicelluloses during the HTP process. The glucose yield decreased coupling without HMF produced when prolonging the reaction time at 120 and 140 °C, which may be ascribed to the occurrence of rehydration reaction at low temperatures [34]. However, it decreased first and then increased when the temperature was up to 160 °C. More interestingly, the content of glucose in corncob residues increased first and then decreased at 160 and 180 °C, respectively. In summary, the following results could be obtained by these observations: (1) The glucose rehydration rate was higher than the cellulose hydrolysis rate at mild conditions (≤140 °C), thus leading to the decreasing of glucose yield. (2) Longer reaction time at high temperatures (≥160 °C) lead to the cellulose hydrolysis reaction, which resulted in the unstable trend of glucose yield.

Since hemicelluloses are the major biomass components released by the HTP process, xylose oligomers (XOS) are other primary substances in hydrolysates. XOS are generally considered as oligosaccharides containing two to ten xylose molecules linked by *β*-1-4 glycosidic bonds [35]. Due to the lack of the standards with the degree of polymerization (DP) ≥7, the amount of each sample was reported up to DP 6 and the results are shown in Table 1. The contents of xylobiose (X2), xylotriose (X3), xylotetraose (X4), xylopentaose (X5) and xylohexose (X6) in hydrolysates steadily increased with increasing reaction time from 0 to 120 min at 120 and 140 °C, respectively. This phenomenon indicated that the hydrolysis of hemicelluloses was smooth in mild conditions. However, when the temperature was up to 160 °C, the basic trends of the XOS contents increased first and then decreased, which was due to the continued hydrolysis of XOS into smaller oligosaccharides or monosaccharide in harsher environments. The highest total xylose content of monosaccharide and XOS (DP ≤ 6) in hydrolysates was obtained at 160 °C for 60 min (272.06 mg g^−1^).

It is believed that the dissolution of hemicelluloses is controlled by their chain length; namely, whether hemicelluloses could be presenting in the liquor depends on their molecular weights [18]. In this paper, lower molecular weight polymers were separated from hydrolysates by the ethanol precipitation procedures demonstrated in the “Methods” section, and the results of their molecular weights and compositions are shown in Table 3.

**Table 3:** Weight-average (*M*_w_), number-average (*M*_n_) molecular weights, polydispersity (*M*_w_ */M*_n_), and monosaccharide compositions (wt%) of ethanol-precipitated fractions

Temp. (°C)	Time (min)	*M*_n_ (g mol^−1^)	*M*_w_ (g mol^−1^)	Glucose (%)	Xylose (%)	Galactose (%)	Arabinose (%)	Glucuronic acid (%)	Xylose/arabinose	Total sugars
120	90	3559	9338	11.60	47.99	4.02	4.52	0.51	10.62	68.64
120	120	1830	6529	10.88	48.23	3.72	4.50	0.76	10.72	68.09
140	30	3358	6414	9.84	55.32	3.22	4.63	0.18	11.95	73.19
140	60	2511	5707	9.75	56.09	2.00	3.03	0.21	18.51	71.08
140	90	2487	5157	8.54	57.56	1.86	2.14	0.22	26.90	70.32
140	120	1637	2690	8.53	50.53	0.83	2.00	0.36	25.27	62.25
160	0	2502	8601	17.14	52.55	5.48	6.50	0.20	8.08	81.88
160	30	2338	2519	13.21	34.50	1.57	1.38	0.22	25.00	50.88
180	0	5252	11,950	7.95	47.22	2.04	3.27	0.12	14.44	60.60
180	5	6571	9408	5.92	13.13	0.74	0.62	0.10	21.18	20.51
180	10	2920	5491	5.50	6.76	0.65	0.37	0.04	18.27	13.32

As shown in Table 1, with the dissolution and depolymerization of hemicelluloses, no ethanol-precipitated hemicelluloses (EPH) could be obtained within short treatment time at 120 °C (<90 min) and 140 °C (<30 min). However, the general trends of the extraction yields at different temperatures were similar except 160 °C under the investigated conditions. The yield of the precipitated hemicelluloses increased first, and then decreased linearly and even no precipitates could be observed, which was ascribed to the further depolymerization of the polymers into oligosaccharides and monosaccharide [18]. Different phenomenon at 160 °C may be due to the long interval of the pretreatment time at the relatively high temperature.

**Fig. 2:**
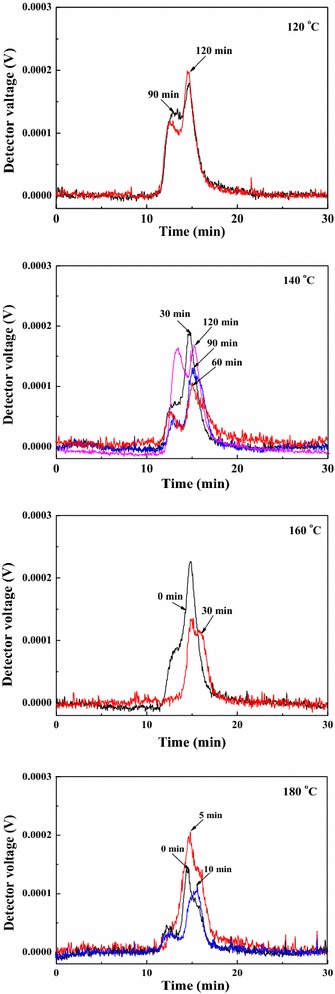
Molecular weight distribution of precipitates isolated from hydrolysates

The weight-average (*M*_w_) and number-average (*M*_n_) molecular weights of the precipitates and their distribution were determined by gel permeation chromatography (GPC) techniques and the results are shown in Table 3 and Fig. 2. All of the fractions exhibited low *M*_w_ ranging between 2519 and 11,950 g mol^−1^, representing the average degree of polymerization (DP_w_) ranging between 19 and 90. The *M*_w_ of the precipitates decreased as the increase of pretreatment time, which was ascribed to the further hydrolysis to a lower degree of polymerization (DP) of the dissolved oligomers. However, when the temperature was up to 160 °C, the *M*_w_ of the precipitates increased as increasing the treatment temperature (160 °C for 0 min, and 180 °C for 0 min), indicating that higher molecular weight hemicelluloses corresponding with a more branched fraction could be obtained at higher temperature and short reaction time in our studies [36]. The trend of *M*_n_ was similar to that of *M*_w_.

The monosaccharide compositions of the EPH are also presented in Table 3. The dominating monomeric sugar components of EPH were identified as xylose, followed by glucose, arabinose and galactose, while glucuronic acid was a trace constituent. These sugar compositions suggested that the EPH were mainly potential galactoarabinoxylan-type polysaccharides and glucuronoarabinoxylans. The ratio of xylose to arabinose is indicative of the degree of linearity or branching of hemicelluloses. The xylose/arabinose ratio in the EPH continuously increased as increasing pretreated temperature or time, suggesting that long chain polymers with small amounts of branching with other monosaccharide compositions could be obtained in relatively higher treatment severity [30]. However, it decreased when prolonging the treatment time up to 120 min at 140 °C and 10 min at 180 °C, which was due to the degradation of the backbone. Opposite trend was observed for the total sugar amounts. The total sugar components of EPH decreased continuously with increasing temperature or time, which was due to the co-precipitation of lignin in acid conditions. Based on these results, it may be inferred that the majority of lignin in the precipitates is lignin–carbohydrate complex, which is called LCC-lignin [37]. The content of lignin in the ethanol-precipitated hemicelluloses increased with the decrease of the hydrolysates’ pH, which may impede the dissolution and degradation of hemicelluloses due to the mass transfer effects. This result was also corresponding to the decrease of the total xylose content of monosaccharide and oligosaccharides at harsh conditions (Table 1) [18].

**Fig. 3:**
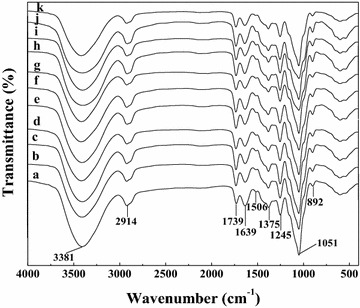
FT-IR spectra of the ethanol-precipitated hemicelluloses from hydrolysates: (*a*) hydrolysis at 120 °C for 90 min, (*b*) hydrolysis at 120 °C for 120 min, (*c*) hydrolysis at 140 °C for 30 min, (*d*) hydrolysis at 140 °C for 60 min, (*e*) hydrolysis at 140 °C for 90 min, (*f*) hydrolysis at 140 °C for 120 min, (*g*) hydrolysis at 160 °C for 0 min, (*h*) hydrolysis at 160 °C for 30 min, (*i*) hydrolysis at 180 °C for 0 min, (*j*) hydrolysis at 180 °C for 5 min, (*k*) hydrolysis at 180 °C for 10 min

The FT-IR spectra of the EPH with different treatment temperature and time are shown in Fig. 3, and the peak assignments are conducted according to the literatures [18, 30, 36]. All samples give similar characteristic bands, which indicate that the structures of EPH have no big change after treated at different temperature and/or time. The band at 892 cm^−1^ is assigned to C_1_ group frequency or ring frequency, indicating the existence of *β*-glucosidic linkage between the xylopyranose units in the main xylan chains [30]. An intense band at 1051 cm^−1^ is respected to the stretching and bending vibrations of C–O, C–C, and C–OH and the glycosidic C–O–C of hemicelluloses [30]. Bands at 1375 and 1245 cm^−1^ are due to the C–H stretching and OH or C–O bending vibration [30]. A small signal at 1506 cm^−1^ arising from the C=C stretching of the lignin aromatic ring reveals the presentation of lignin in EPH. The band at 1639 cm^−1^ is presumed to be due to the water-related absorbance, while the band at 1739 cm^−1^ is corresponding to the carbonyl stretching assigned to the acetyl, glucuronic acid and ferulic ester groups of polysaccharides [36]. Bands at 3381 and 2914 cm^−1^ are due to the stretching vibrations of OH and CH, respectively [36].

### Heterogeneous conversion of hemicellulose-derived sugars over Sn-MMT in the biphasic system

**Fig. 4:**
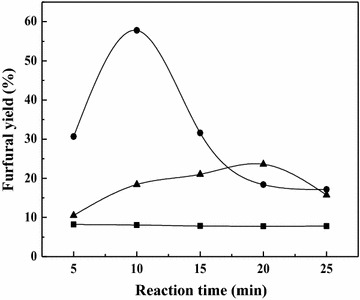
Furfural yields of different hydrolysates. *Filled square* pretreated temperature: 180 °C, pretreated time: 15 min; *filled triangle* pretreated temperature: 160 °C, pretreated time: 60 min; *filled circle* pretreated temperature: 160 °C, pretreated time: 90 min

To understand the relationship between the hydrolysate composition and the furfural yields, three specific samples with the maximum xylose content (160 °C, 90 min), the maximum xylobiose (X2) content (180 °C, 15 min), and the maximum total xylose content in monosaccharide and oligosaccharides (DP ≤ 6) (160 °C, 60 min) were selected for the following experiments. Our group previously found that Sn-MMT had the excellent catalytic performance in the conversion of xylose into furfural in the SBP/NaCl-DMSO system [20]. However, no detailed and systematic studies were displayed for the furfural production from corncob hydrolysates by a two-step process. In the present work, the obtained hydrolysates from the microwave-assisted hydrolysis of corncob were subjected to further hydrolysis and dehydration to yield furfural using Sn-MMT as a solid catalyst in a biphasic system (SBP/NaCl-DMSO) as the second stage of the process and the results are presented in Table 4 and Fig. 4.

**Table 4:** Composition of products (mg per gram sample, mg g^−1^) after the heterogeneous reaction

Treated temp. (°C)	Treated time (min)	Reaction time (min)	Glucose	Xylose	Arabinose	Formic acid	Acetic acid
160	60	5	1.96	38.72	1.59	0.36	2.73
160	60	10	3.79	32.40	1.42	0.46	3.18
160	60	15	4.17	30.15	1.36	0.53	3.18
160	60	20	4.43	26.53	0.83	0.56	3.35
160	60	25	4.68	21.46	0.73	0.59	2.96
160	90	5	5.39	13.45	0.82	0.78	3.27
160	90	10	5.72	10.66	0.47	0.88	3.44
160	90	15	5.15	9.01	0.48	0.88	3.72
160	90	20	4.92	7.04	0.32	0.96	3.41
160	90	25	4.52	6.51	0.23	0.97	3.33
180	15	5	3.40	18.60	0.19	0.43	2.48
180	15	10	3.82	10.46	0.13	0.56	2.96
180	15	15	4.17	7.75	0.01	0.68	2.79
180	15	20	4.46	3.43	0.00	0.76	2.71
180	15	25	6.10	1.98	0.00	0.91	2.61

Reaction conditions: hydrolysates (3 mL, saturated with NaCl), Sn-MMT (0.1 g), DMSO (1 mL), SBP (4 mL), 190 °C

Experiments for the conversion of hydrolysates to furfural were performed in an autoclave using the drying oven as the heating source, since they are common and provide moderate heating to avoid the rapid degradation of the target products. During the furfural production process, XOS and polymers are hydrolyzed to form monomeric pentoses first, followed by dehydrating them to yield furfural. As observed in Table 4, hydrolysates employed in this study with the highest total xylose content of monomeric and oligosaccharides (160 °C, 60 min) resulted in the highest monomeric pentose yields. It should be noted that the xylose yield obtained here was lower than that in the first stage, which suggested that the dehydration reactions were much faster than the hydrolysis reactions. The highest furfural yield (57.80 %) was obtained at 190 °C after 10 min from the hydrolysates with the highest monomeric pentose content conducted at 160 °C for 90 min in the first process. This yield is only slightly better than the one we reported previously (54.15 % from water-soluble fraction of corncob treated at 140 °C for 5 h) [20], which may be due to the different molecular weights of the oligosaccharides in the hydrolysates. In addition, the decrease in treatment time also can lead to the reducing consumption of energy. However, the hydrolysate with the highest xylobiose (X2) as well as the second largest total xylose content (180 °C, 15 min) has the lowest furfural yield (Fig. 4). These results indicated that the production of furfural has a direct connection with the monomeric pentose content in the hydrolysates.

Controlled experiments were carried out to investigate the influence of the initial xylose concentration of the hydrolysates on the furfural production, and the results are shown in Table 5. Compared with the hydrolysates (Table 5, entry 1), lower furfural yield was obtained using the pure xylose solution as the reagent (Table 5, entry 2), which presented the same initial xylose concentration in the hydrolysates. Moreover, the furfural yield from the pure xylose solution was much lower than that from the hydrolysates with the same total xylose amount in monosaccharide and oligosaccharides (DP ≤ 6) (Table 5, entry 3), which may be ascribed to the reason that the slow release of pentose monomers from the oligomers can impede the formation of humins. These phenomenons were in good agreement with the hypothesized that furfural yields from biomass hydrolysates may exceed those from pure xylose solution [38]. In addition, the yield of furfural decreased with the increment of the initial xylose content in the hydrolysates (Table 5, entries 1, 4 and 5). This is because the condensation loss reaction easily occurs in higher xylose concentration, leading to the formation of furfural–pentose [38].

**Table 5:** Comparison of the hydrolysates and the pure xylose solution

Entries	Samples	Xylose concentration (%)	Furfural yield^a^ (%)
1	Hydrolysates^b^	1.00	76.09
2	Pure xylose solution^c^	1.00	72.42
3	Pure xylose solution^c^	2.29	51.13
4	Hydrolysates^b^	2.00	44.75
5	Hydrolysates^b^	5.00	26.06

^a^Furfural yield: based on the total xylose content of monomeric and xylooligosaccharides in the hydrolysates

^b^Reaction conditions: hydrolysates (3 mL, pretreated temperature: 160 °C, pretreated time: 90 min, saturated with NaCl), Sn-MMT (0.1 g), DMSO (1 mL), SBP (4 mL), 190 °C, 10 min

^c^Reaction conditions: 3 mL saturated NaCl solution, Sn-MMT (0.1 g), DMSO (1 mL), SBP (4 mL), 190 °C, 10 min

An advanced and comprehensive hydrolysis approach is imperative for the biomass-to-furfural technologies. To improve furfural yields in an eco-friendly and effective manner, we will couple this approach with ball milling with the small amount of dilute acid before the experiments in our future study, which will produce higher yield of monomeric sugar in the first pretreatment stage.

## Conclusions

A two-step process was proposed to yield furfural from corncob using microwave-assisted HTP as the first stage and heterogeneously catalyzed conversion as the second stage. The dissolution and depolymerization of sugars from corncob and the relationship between the hydrolysate composition and the furfural yields were investigated.

In the first stage of the process, the released hemicelluloses from corncob were mainly in forms of monosaccharide, oligosaccharides, and water-soluble polysaccharides. About 86.67 % yield of xylose presented as monosaccharide and oligosaccharides (DP ≤ 6) after the microwave-assisted HTP at 160 °C for 60 min. A positive effect of microwave-assisted HTP on producing sugars was observed during this stage.

During the second stage, hydrolysates with the maximum xylose content (160 °C, 90 min), the maximum xylobiose (X2) content (180 °C, 15 min), and the maximum total xylose content in monosaccharide and oligosaccharides (DP ≤ 6) (160 °C, 60 min) were further conducted to produce furfural using Sn-MMT as the catalyst in the SBP/NaCl-DMSO system, respectively. The highest furfural yield (57.80 %) was obtained from the hydrolysates with the maximum xylose content. Moreover, controlled experiments showed that furfural yields from corncob hydrolysates were higher than those from the pure xylose solutions, and lower initial xylose concentration may be in favor of the furfural production. These results indicated that the production of furfural from biomass raw materials can be controlled by the depolymerization of hemicelluloses.

Further studies are in progress for improving the furfural yields coupling this approach with ball milling with the small amount of dilute acid before the experiments and the subsequent exploitation of the solid residues as the raw materials for the ethanol production in the first stage.

## Methods

### Experimental materials

Acetone (≥99.0 %, AR), ethanol (≥99.0 %, AR), H_2_SO_4_ (≥98.0 %, AR), NaH_2_PO_4_ (≥99.0 %, AR), Na_2_HPO_4_ (≥99.0 %, AR), SnCl_4_·5H_2_O (≥99.0 %, AR), montmorillonite (≥98.0 %, GR) and NaCl (≥99.5 %, AR) were purchased from Kermel Co. Ltd. (Tianjin, China). DMSO (≥99.0 %, AR) obtained from Lingfeng Chemical Reagent Co. Ltd. (Shanghai, China) was used in all DMSO co-solvent reactions. SBP (≥97.0 %, GC) was purchased from Tokyo Chemical Industry (Japan) and used as the extracted solvent in the furfural production process. Standard reagents of xylose (≥99.0 %, HPLC), glucose (≥99.0 %, HPLC), arabinose (≥98.0 %, HPLC), galactose (≥99.0 %, HPLC), formic acid (50 %, HPLC), acetic acid (≥99.9 %, HPLC), glucuronic acid (≥99.0 %, HPLC), furfural (≥99.0 %, HPLC) and 5-hydroxymethylfurfural (≥99.0 %, HPLC) were purchased from Sigma-Aldrich. Xylobiose (≥95.0, HPLC) and xylotriose (≥95.0 %, HPLC) were purchased from WAKO (Japan). Xylotetraose (≥95.0 %, HPLC), xylopentaose (≥90.0 %, HPLC) and xylohexaose (≥90.0 %, HPLC) were obtained from Megazyme (Ireland). All reagents were used without further purification.

Corncob was obtained from a farm in Shandong Province (China). Prior to the experiments, it was ground to pass through 40 and 80 mesh screens and then extracted with the volume ratio of acetone to ethanol (2:1) for 6 h in a soxhlet extractor. The extracted solid was washed with water and oven-dried to constant weight at 55 °C. Thereafter, corncob was milled for 6 h (400 rpm min^−1^) by a ball-milling machine and then stored in desiccators. Biomass composition of the corncob after extraction was determined according to the established National Renewable Energy Laboratory procedure (NREL/TP-510-42618) with a resulting composition 37.09 wt% of glucose, 31.39 wt% of xylose, 2.28 wt% of arabinose and 14.47 wt% of lignin.

### Microwave-assisted hydrothermal pretreatment

A closed-vessel microwave oven (100 mL, GAS-800, Beijing Xiang-Hu Science and Technology Development Reagent Co. Ltd., China) equipped with a magnetic stirrer, a temperature probe and an autoclave was used for the HTP experiments in this study. In all cases, corncob was first suspended in DI water with a solid to liquor ratio of 1:10 (g mL^−1^) and ultrasound for 30 min. A microwave irradiation power of 600 W was applied to heat the suspension to 120 °C in 3 min and then kept for 2 min. Then, the temperature was raised with a heating rate of 20 °C in every 2 min and a retention time of 2 min in every 20 °C interval. It is important to note that this heating way can provide a buffer stage to avoid the over-heating under 600 W irradiation power and alleviate the side reactions such as the dehydration and rehydration of monosaccharide. Reaction time counted from zero when the temperature reached to the desired point. After the reaction, the autoclave was natural cooling to room temperature. Thereafter, the pretreated corncob substrate and the hydrolysate were separated by filtration for further analysis and experiments.

### Precipitation of ethanol-insoluble hemicelluloses from hydrolysates

After the microwave-assisted HTP process, the hydrolysate was added into four times volume ethanol to precipitate the ethanol-insoluble hemicelluloses. The sediment was washed with acid ethanol (pH = 5.5–6.0) for several times and then freeze-dried.

### Conversion of hydrolysates over tin-loaded montmorillonite in the biphasic system

Details on the synthetic procedures and characterizations of Sn-MMT had been published in our previous study [20]. Experiments for the conversion of hydrolysates to furfural were carried out in an autoclave (25 mL, Henan Yuhua Co. Ltd., China) using Sn-MMT as the catalyst in the SBP/NaCl-DMSO system. In brief, the hydrolysate was saturated with the solid NaCl and the upper clean liquid was obtained as the reagent. Sn-MMT and organic solvents (DMSO and SBP) were added in a desired ratio, respectively. The autoclave was heated up to the desired temperature with a heating rate of 2 °C min^−1^ and kept for a required time. Initial reaction time (*t* = 0) was taken when the temperature set point was reached. After the reaction, the reactor was cooled quickly to room temperature in ice water bath, and the products were separated into water phase and organic phase with the separating funnel for further analysis.

### Analytical methods

The composition of hydrolysates was measured by high-performance liquid chromatography (HPLC) equipped with a refractive index detector (Waters, USA) and a Bio-rad Aminex^®^ HPX-87H (300 × 7.8 mm) column (Bio-rad, USA). H_2_SO_4_ (5 mM) was employed as the mobile phase with a flow rate of 0.5 mL min^−1^ at 50 °C. Calibration curves were performed with a standard solution of glucose, xylose, arabinose, formic acid, acetic acid, furfural and HMF, respectively.

Xylooligosaccharides in hydrolysates were detected by HPLC using a Hi-Plex Na column (7.7 × 300 mm, Agilent, USA) in combination with a refractive index detector (Waters, USA). DI water was employed as the mobile phase with a flow rate of 0.2 mL min^−1^ at 80 °C. Calibration curves of xylobiose (X2), xylotriose (X3), xylotetraose (X4), xylopentaose (X5) and xylohexose (X6) were established for the quantitative calculation.

High-performance anion-exchange chromatography (HPAEC, Dionex ICS-3000, Sunnyvale, CA, USA) coupled with a Carbopac™ PA-20 column (4 × 250 mm, Dionex, USA) was used to determine the monosaccharides and uronic acids that liberated from the precipitated hemicelluloses by the hydrolysis with H_2_SO_4_ (1 M) at 105 °C for 2.5 h. Detailed procedure was illustrated in the Ref. [36]. Calibration curves were performed with a standard solution of glucose, xylose, galactose, arabinose and glucuronic acid, respectively.

The molecular weights of the precipitated ethanol-insoluble hemicelluloses were determined by gel permeation chromatography (GPC) with an Agilent PL aquagel-OH MIXED-H column (300 × 7.5 mm) and an evaporative light-scattering detector (Wyatt, USA). Sodium phosphate buffer (5 mM, pH 7.5) containing NaCl (0.02 N) was employed as the eluent with a flow rate of 0.5 mL min^−1^ at 35 °C.

The composition of the solid residue was measured according to the National Renewable Energy Laboratory’s Laboratory Analytical Procedures (NREL/TP-510-42618). Briefly, sample (300 mg) was hydrolyzed with 72 % H_2_SO_4_ (3.0 mL) at 30 °C for 1 h. The hydrolysate was diluted with DI water (84 mL), followed by autoclaving for 1 h at 121 °C. Carbohydrates were analyzed by HPLC as described above and the acid-soluble lignin was measured by ultraviolet spectrophotometer (UV-1800, Shimadzu, Japan). Experiments were duplicated to decrease the errors.

The FT-IR spectra of the pretreated corncob and the precipitated ethanol-insoluble hemicelluloses were obtained on a spectrophotometer (Tensor 27) using a KBr disk containing 1 % of the finely ground sample. Scanning electron microscopy (SEM) was used to analyze the surface morphology characterization of the samples (S-4300, Hitachi, Japan). X-ray diffraction (XRD) patterns of samples were recorded on a Bruker diffractometer with Cu K*α* radiation. The tube voltage was 40 kV and the current was 40 mA. The selected 2*θ* range was 5°–40°, scanning at a step of 0.02°.

The monosugars, organic acids and furfural contents of the liquid products after the heterogeneously catalyzed conversion were detected by HPLC as described above. Furfural yield was defined by the following formula:

$${\text{Furfural}}\;{\text{yield}} = \frac{{{\text{moles}}\;{\text{of}}\;{\text{produced}}\;{\text{furfural}}}}{{{\text{moles}}\;{\text{of}}\;{\text{xylose}}\;{\text{in}}\;{\text{corncob}}}} \times 100\%$$

## Additional file

Additional file 1:
**Figure S1**. SEM images of the treated corncob. **Figure S2**. XRD patterns of the hydrothermal treated corncobs.

## Abbreviations

HTP: hydrothermal pretreatment
Sn-MMT: tin-loaded montmorillonite
SBP: 2-sec-butylphenol
DMSO: dimethyl sulfoxide
HMF: 5-hydroxymethylfurfural
LCMs: lignocellulosic materials
XOS: xylose oligomers
DP: degree of polymerization
X2: xylobiose
X3: xylotriose
X4: xylotetraose
X5: xylopentaose
X6: xylohexose
EPH: ethanol-precipitated hemicelluloses
LCC-lignin: lignin–carbohydrate complex
*M*_w_: weight-average molecular weight
*M*_n_: number-average molecular weight
NREL: National Renewable Energy Laboratory
FT-IR: Fourier-transform infrared
GPC: gel permeation chromatography
HPLC: high-performance liquid chromatography
HPAEC: high-performance anion-exchange chromatography
SEM: scanning electron microscopy
XRD: X-ray diffraction
