# Supplementary material for: Functional relationship of furfural yields and the hemicellulose-derived sugars in the hydrolysates from corncob by microwave-assisted hydrothermal pretreatment
Source: Biotechnol Biofuels. 2015 Aug 27;8:127. doi: 10.1186/s13068-015-0314-z (PMC4549872; doi:10.1186/s13068-015-0314-z)
Supplement: Additional file 1: — Figure S1. SEM images of the treated corncob. Figure S2. XRD patterns of the hydrothermal treated corncobs. [file 13068_2015_314_MOESM1_ESM.pdf]

Figure Captions

Figure S1 SEM images of the treated corncob

Figure S2 XRD patterns of the hydrothermal treated corncobs

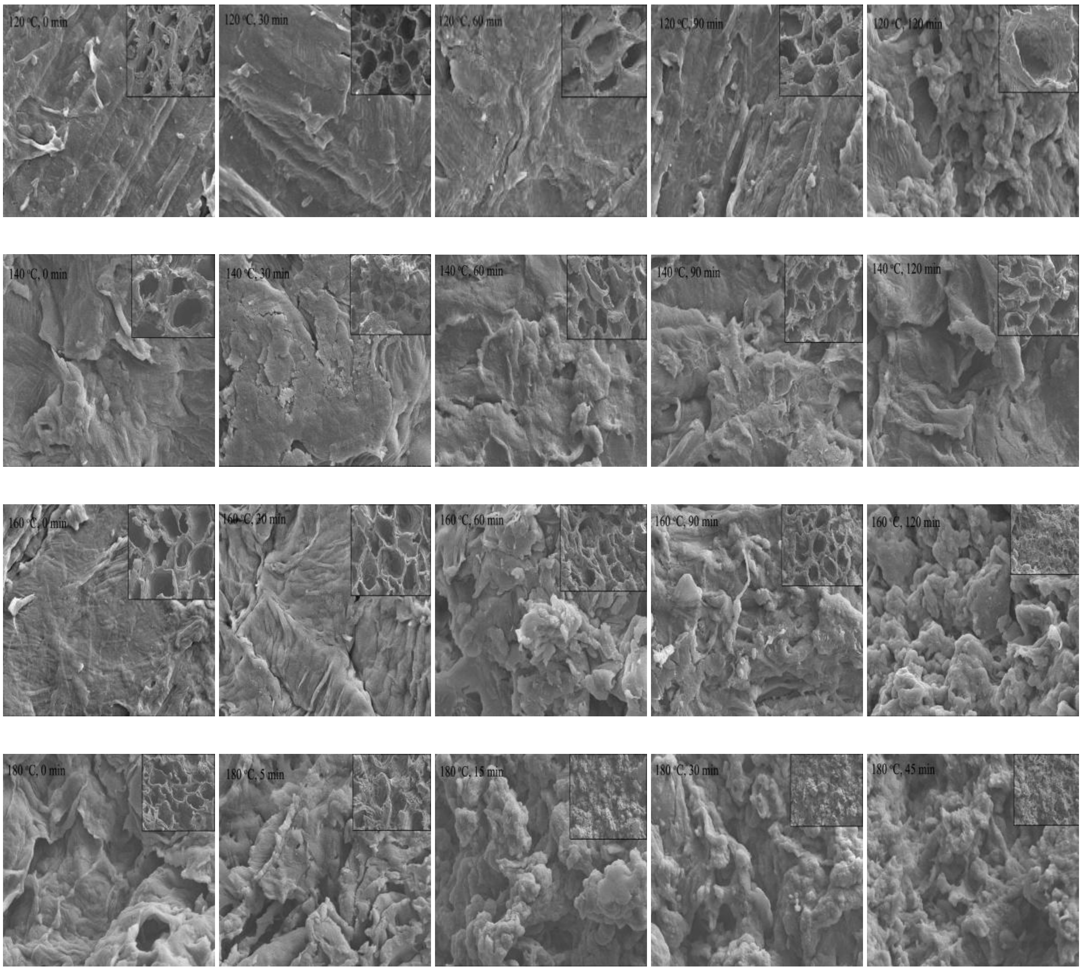
120 °C, 0 min
120 °C, 30 min
120 °C, 60 min
120 °C, 90 min
120 °C, 120 min
140 °C, 0 min
140 °C, 30 min
140 °C, 60 min
140 °C, 90 min
140 °C, 120 min
160 °C, 0 min
160 °C, 30 min
160 °C, 60 min
160 °C, 90 min
160 °C, 120 min
180 °C, 0 min
180 °C, 5 min
180 °C, 15 min
180 °C, 30 min
180 °C, 45 min

Figure S1

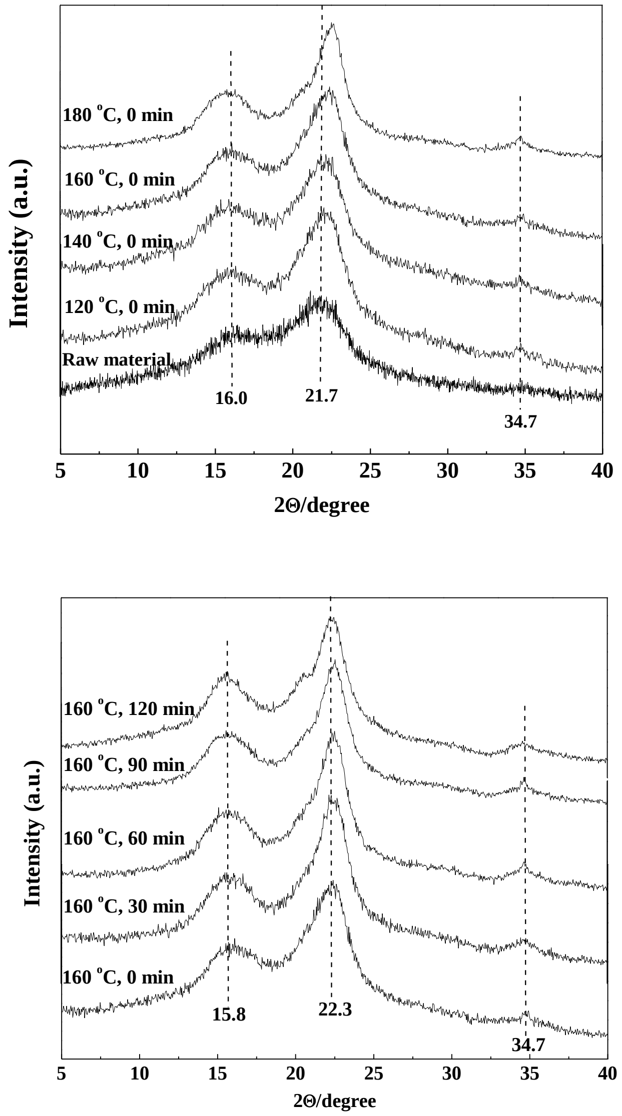
180 °C, 0 min
160 °C, 0 min
140 °C, 0 min
120 °C, 0 min
Raw material
16.0
21.7
34.7
Intensity (a.u.)
5
10
15
20
25
30
35
40
2Θ/degree
160 °C, 120 min
160 °C, 90 min
160 °C, 60 min
160 °C, 30 min
160 °C, 0 min
15.8
22.3
34.7
Intensity (a.u.)
5
10
15
20
25
30
35
40
2Θ/degree

Figure S2
